# Comparison of wide field optical coherence tomography angiography with extended field imaging and fluorescein angiography in retinal vascular disorders

**DOI:** 10.1371/journal.pone.0214892

**Published:** 2019-04-09

**Authors:** Marco Pellegrini, Mariano Cozzi, Giovanni Staurenghi, Federico Corvi

**Affiliations:** Eye Clinic, Department of Biomedical and Clinical Science “Luigi Sacco”, Sacco Hospital, University of Milan, Milan, Italy; Massachusetts Eye & Ear Infirmary, Harvard Medical School, UNITED STATES

## Abstract

**Purpose:**

To compare swept source OCTA device, with and without the extended field imaging (EFI) technique, to standard fluorescein angiography (FA) in the clinical practice.

**Methods:**

Consecutive patients with vascular disorder patients underwent FA with 55-degree lens (Spectralis Heidelberg Engineering, Heidelberg, Germany) and OCTA with the prototype PlexElite (Carl Zeiss Meditec, Dublin, CA) using a 12 mm x 12 mm volume scan pattern centered on the fovea and a prototype of + 20.00-diopter designed specifically by Zeiss. The imaging methods were compared for visible field of view, extension of non-perfused areas, presence and number of neovessels, vessel density (VD) and fractal dimension (FD).

**Results:**

Forty-three eyes of 27 patients were included. The mean extension ratio of EFI SS-OCTA compared to SS-OCTA without EFI and FA were 1.97 ± 0.02 and 0.85 ± 0.01. The mean extension of non-perfused areas with EFI SS-OCTA (34.22 ± 33.4 mm^2^) was significantly higher than SS-OCTA without EFI (20.46 ± 18.70 mm^2^), and with FA (27.55 ± 4.4 mm^2^). The mean VD and FD of EFI SS-OCTA were significantly different compared to SS-OCT without EFI.

**Conclusions:**

EFI SS-OCTA captured larger areas than SS-OCTA without EFI and FA. OCTA in a single shot is able to obtain more information of the retina without the use of montage techniques. Despite the determination of retinal ischemia seems to be easier and more accurate using EFI SS-OCTA, FA offers more details of the perfusion status of the retina.

## Introduction

In clinical practice the gold standard for the evaluation of retinal perfusion and neovascularization is fluorescein angiography (FA) [1]. It is based on the intravenous injection of fluorescein dye followed by images acquisition. However, FA has several limitations including possible side effects ranging from nausea, vomiting, yellow pigmentation to severe anaphylactoid reactions [2]. Moreover, it produces only two-dimensional images in which fluorescence signals of the superficial and deep capillary networks overlap and are difficult to distinguish, even more when the dye leaks [1,3].

The development of optical coherence tomography angiography (OCTA) has provided a new noninvasive dyeless imaging technique to represent retinal vessels [4,5]. In contrast from FA, it provides en-face images of different retinal plexuses to perform qualitative and quantitative analysis as vessel density (VD) and fractal dimension (FD) [6,7]. Nevertheless, the application of this novel imaging tool in the assessment of peripheral vascular abnormalities is quite controversial due to its primary application to posterior pole.

Recently, it has been described a new imaging technique for extending the scan length of OCT [8]. A convex lens of + 20.00-diopter is placed between the eye and an OCT probe increasing theoretically the imaging light incidence angle and resulting in extended field imaging (EFI).

The use of OCTA with an EFI technique may be used in the clinical practice to evaluate the retinal perfusion status in patients with vascular disorder. For this reason, the purpose of our study was to test the feasibility of swept source OCTA device, with and without the EFI technique and to compare this imaging technique to conventional FA.

## Material and methods

This study was approved by the Luigi Sacco Hospital Ethics Committee in Milan. A written informed consent was obtained from all subjects. The study followed the tenets of the Declaration of Helsinki for research involving human subjects. Consecutive patients with vascular disorder presenting to the Eye Clinic, Department of Biomedical and Clinical Sciences, Luigi Sacco Hospital, University of Milan from September 15^th^ 2017 to March 15^th^ 2018 were prospectively included. Inclusion criteria were age ≥ 18 years old and the presence of either retinal or choroidal vascular disorders. Subjects with significant media opacities and poor cooperation were excluded from the study. All subjects underwent a complete ophthalmic examination, including a detailed medical history, refraction, intraocular pressure measurement, anterior segment and dilated fundus examination and FA performed using the 55-degree lens (Spectralis Heidelberg Engineering, Heidelberg, Germany). The same day, OCTA imaging was performed using the PlexElite (Version 1.6.0.21130, Carl Zeiss Meditec, Dublin, CA). An expert operator (MC) performed all the examinations using a 12 mm x 12 mm volume scan pattern centered on the fovea and using a + 20.00-diopter add-on prototype lens designed specifically by Zeiss to perform EFI examination and to avoid the positional instability of the lens. Following each examination, images were deemed acceptable if the retinal vessels were clearly visible and distinguishable.

### Image analysis

The field of examination by EFI-OCTA was analyzed for each eye and compared with standard 55-degree field FA. Horizontal and vertical distances between two landmark points were measured using ImageJ software (National Institutes of Health, Bethesda, Maryland, USA), as previously described [9–11]. The overall expansion of the field of view was then computed by multiplying the horizontal and vertical extension ratio values.

Vessel density was calculated using ImageJ (National Institutes of Health [NIH], Bethesda, Maryland, USA) [10,12]. In particular, image from 8-bit was split into the three channels (red, green and blue) using the red channels as the reference. Image was adjusted by the “mean threshold” algorithm which automatically computes the threshold value as the mean of the local grayscale distribution. Vessel density was calculated as the ratio of the area occupied by vessels divided by the total area.

**Fig 1:**
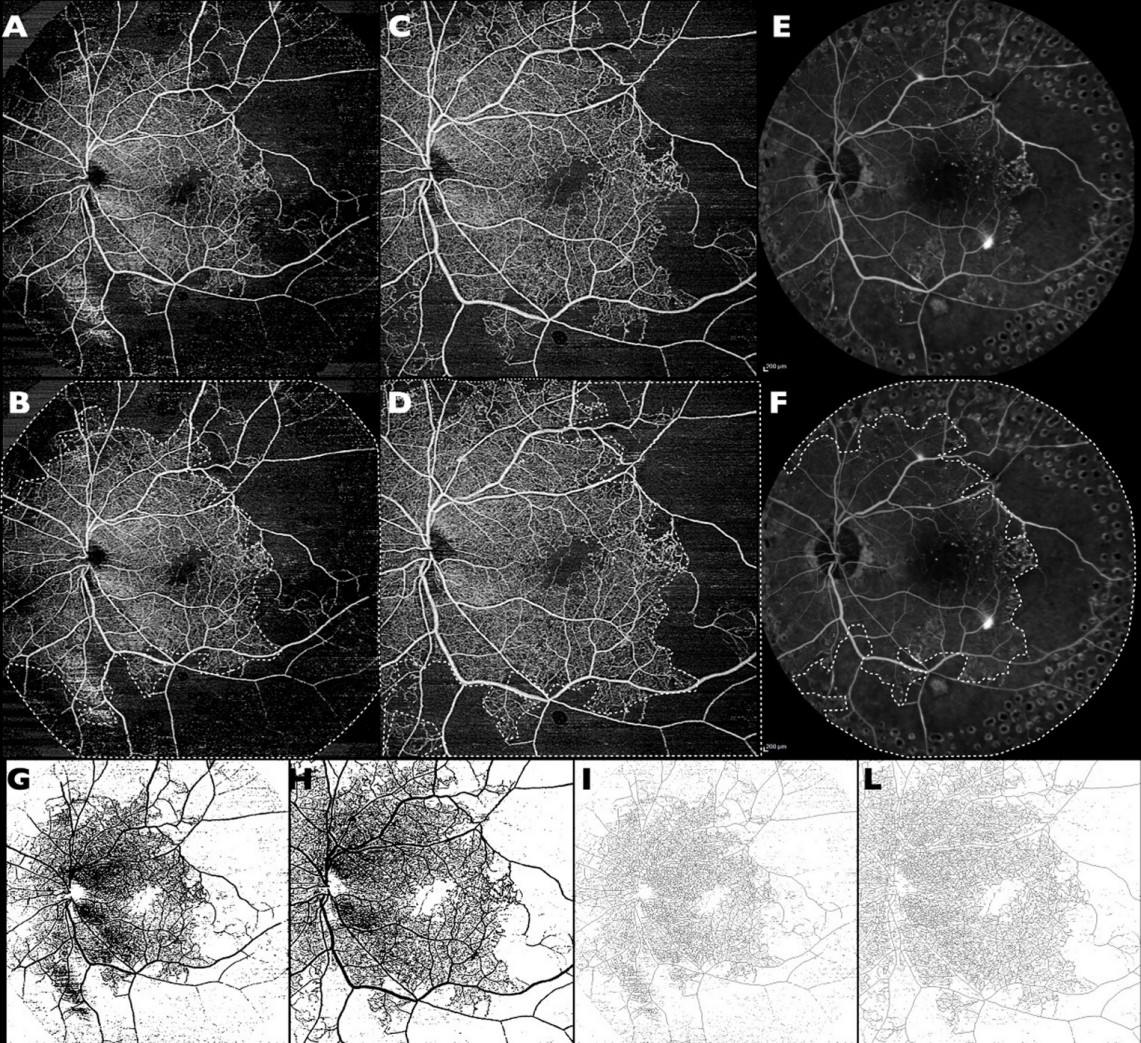
Swept-source optical coherence tomography angiography (SS-OCTA) and fluorescein angiography (FA) images of a representative eye with diabetic retinopathy. Extended field imaging (EFI) SS-OCTA (A and B),12 mm x 12 mm SS-OCTA without EFI (C and D), FA with 55 degrees lens (E and F), the corresponding binarized images (G and H) and fractal dimension images (I and L). White dot line delimits the areas of non-perfusion.

Consequently, the binarized image was skeletonized and FD was calculated using Fractalyse (TheMA, Besanc on Cedex, France) [12,13]. It is an index of the branching complexity of capillary network ranging from 0 to 2. Lower FD values correspond to a low complexity of vessel branching, while higher values correspond to a more complexity (Fig 1).

The extension of retinal non perfused areas was evaluated by 2 graders (FC and MP) which measured manually and masquerade to the clinical background. Each image was displayed to the graders in casual distribution. FA images used for delineation of the extension of retinal non-perfusion were taken in the early phases, within 1 minute after dye injection, in order to reduce the effect of the leakage of the dye. Moreover, graders evaluated the presence and number of new blood vessels.

### Statistical analysis

Statistical analysis was performed using SPSS v.22.0 (IBM Corp., Armonk, NY, USA). All values were expressed as the mean ± standard deviation. Data were analyzed using Shapiro-Wilk test to evaluate the normality of sample distribution. The Wilcoxon signed rank test was used to compare the mean values between different groups. A P-value less than 0.05 was considered significant. Pearson’s rank correlation was used to determine the correlation coefficients of continuous variables. The agreement of measurements between readers was assessed using interclass correlation coefficient (ICC).

## Results

A total of 43 eyes of 27 patients (age, 61.3±14.4 years; 17 male and 10 female) were included in this study; 5 eyes of 4 patients were excluded due to image artefacts related to poor fixation. Thirty-two eyes (74%) were affected by diabetic retinopathy, 8 (19%) by retinal vein occlusion, 2 (5%) by retinal vein occlusion secondary to radiation retinopathy and 1 (2%) by Coat’s disease. Twenty-five eyes (58%) were treatment naïve whereas 18 (42%) underwent previous treatments including laser photocoagulation or with anti-vascular endothelial growth factor (VEGF) injections in the previous 6 months. The mean BCVA was 20/40 (range, 20/200 to 20/20) (Table 1) (S1 File).

**Table 1:** Demographic features.

Feature	Number (%)
Age (years)	61.3 (18–90
Caucasian	27 (100)
Best corrected visual acuity, mean (range)	20/40 (20/200-20/20)
Retinal vascular disease	43 (100)
*DR*	32 (74)
*NPDR*	19 (59)
*PDR*	13 (41)
*RVO*	8 (19)
*RVO secondary to radiation retinopathy*	2 (5)
*Coat’s disease*	1 (2)
Treatment naïve	43 (100)
*Yes*	25 (58)
*No*	18 (42)
Treatment	18 (100)
*Laser photocoagulation*	14 (77)
*Intravitreal anti-VEGF injection*	4 (33)

DR, diabetic retinopathy; NPDR, nonproliferative diabetic retinopathy; PDR, proliferative diabetic retinopathy; RVO, retinal vein occlusion; VEGF, vascular endothelial growth factor.

### Extension of field of view

The mean extension ratio of EFI SS-OCTA compared to SS-OCT without EFI in the horizontal, vertical and overall direction were 1.40 ± 0.02 (range, 1.34–1.43), 1.40 ± 0.01 (range, 1.38–1.43) and 1.97 ± 0.03 (range, 1.91–2.00). The extension ratios in the two dimensions were not significantly different (P = 0.946).

The mean extension ratio of FA compared to EFI SS-OCTA in the horizontal, vertical and overall direction were 0.92 ± 0.01 (range, 0.91–0.96), 0.91 ± 0.01 (range, 0.90–0.93) and 0.85 ± 0.01 (range, 0.82–0.88). The extension ratios in the two dimensions were not significantly different (P = 0.104).

The mean extension ratio of FA compared to SS-OCTA without EFI in the horizontal, vertical and overall direction were 1.30 ± 0.01 (range, 1.27–1.32), 1.29 ± 0.01 (range, 1.26–1.31) and 1.68 ± 0.02 (range, 1.63–1.72). The extension ratios in the two dimensions were not significantly different (P = 0.122).

### Extension of retinal non-perfusion areas and presence of neovascularization

The mean extension of non-perfused areas with EFI SS-OCTA was 34.24 ± 33.3 mm^2^ (range, 0–133.2), with SS-OCTA without EFI 20.42 ± 18.70 mm^2^ (range, 0–74.76) and with FA 27.56 ± 28.15 mm^2^ (range, 0–111.3). The comparisons of mean extension of non-perfused areas between the different techniques were significantly different (SS-OCTA with EFI and without EFI, P < 0.0001; SS-OCTA with EFI and FA, P < 0.0001; SS-OCTA without EFI and FA, P <0.01).

The interobserver agreement of measurement of non-perfused areas between reader for EFI SS-OCTA was 0.879 (95% CI 0.846 to 0.953), for SS-OCTA without EFI 0.914 (95% CI 0.867 to 0.928) and for FA 0.892 (95% CI 0.871 to 0.933).

The specificity and sensitivity for detection of new blood vessels were respectively 97% and 100% for EFI SS-OCTA 100% and 80% and for SS-OCTA without EFI. New blood vessels were found in 25.5% eyes with EFI SS-OCTA, in 18.6% eyes with SS-OCTA without EFI and 23.2% eyes with FA. No differences were found in term of mean number of new blood vessels between FA and EFI SS-OCTA (0.58 ± 1.67 and 0.60 ± 1.67, P = 0.998). No differences were found about the mean number of new blood vessels between FA and SS-OCTA without EFI (0.58 ± 1.70 and 0.51 ± 1.56, P = 0.148). No differences were found about the mean number of new blood vessels between EFI SS-OCTA and without EFI (P = 0.071). The interobserver agreement of detection of neovascularization between reader for EFI SS-OCTA was 0.905 (95% CI 0.862 to 0.948), for SS-OCTA without EFI 0.926 (95% CI 0.893 to 0.959) and for FA 0.967 (95% CI 0.935 to 0.987).

### Vessel density and fractal dimension

The mean vessel density of EFI SS-OCTA was significantly different compared to SS-OCTA without EFI (0.30 ± 0.04, range 0.18–0.38 and 0.32 ± 0.01, range 0.19–0.38 respectively) P < 0.0001. The mean fractal dimension of EFI SS-OCT was significantly different compared to SS-OCTA without EFI (1.69 ± 0.02, range 1.61–1.73 and 1.72 ± 0.02, range 1.63–1.75) P < 0.0001.

Using EFI SS-OCTA, VD was positively correlated to FD (r = 0.841, P < 0.0001), the non-perfusion areas were negatively correlated to VD (r = - 0.452, P = 0.003) and FD (r = - 0.648, P < 0.0001). Using SS-OCTA without EFI, VD was positively correlated to FD (r = 0.765, P < 0.0001), the non-perfusion areas were negatively correlated to VD (r = - 0.501, P = 0.0008) and FD (r = - 0.613, P < 0.0001).

## Discussion

OCTA is a recent imaging technique that allows qualitative and quantitative assessment of retinal and choroidal vessels in a limited area using computer-generated algorithms to determine blood flow [4,5]. One of the main deficiencies of this imaging technique is related to its limited field of view suggesting a primary application to disorders affecting the macular region. However, previous reports have documented the possibility of using additional convex lenses to increase the examination field of view (EFI technique) [8].

In this study we investigated the feasibility of EFI SS-OCTA in clinical practice by comparing its qualitative and quantitative parameters with standard FA and SS-OCTA without EFI. Our results documented that EFI SS-OCTA captured larger areas of the fundus than SS-OCTA without EFI and FA images with 55° field of view. This is a great advantage since EFI OCTA consists in a single fast acquisition able to obtain more informations of the retina without need for montage techniques that necessarily require extra time to obtain multiple scans, overlapping areas and an extra time to remove inaccuracies due to subtle misalignments.

**Fig 2:**
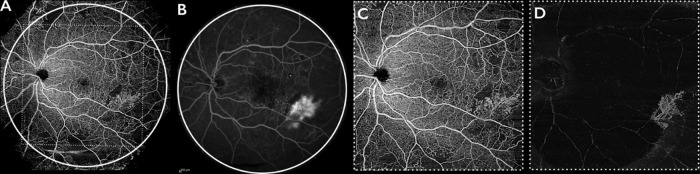
Fluorescein angiography (FA) and SS-OCTA images of one representative eye with diabetic retinopathy. SS-OCTA performed with the EFI technique (A) with the white circle indicating the field of view of FA obtained with the standard Heidelberg 55 degrees lens (B) and white square indicating the field of view of SS-OCTA without EFI (C). The vitreoretinal interface segmentation (D) reveals the new blood vessels in the vitreous cavity.

We also performed a qualitative and quantitative analysis regarding the presence and the number of new blood vessels finding no differences comparing several imaging modalities. In this contest, to evaluate new blood vessels is recommended the use of vitreoretinal interface slab that excludes the retinal vessels and enhances the visualization of new blood vessels protruding in the vitreous cavity as shown in Fig 2.

On the other side, the evaluation of non-perfusion areas revealed a significantly larger extension with EFI SS-OCTA compared to FA and SS-OCTA without EFI. Consequently, the measurement of VD and FD revealed significant lower values with EFI SS-OCTA compared SS-OCTA without EFI. In fact, the extension of non-perfusion areas is negatively correlated with VD and FD, suggesting that the increase of non-perfusion areas is associated with a reduction of VD defined as the area occupied by vessels divided by the total area and consequently of FD. Moreover, VD is positively correlated with FD, suggesting that the increase of VD is associated with the increase of the branching complexity of the capillary network.

Possible explanation of these results are related to the wider field of EFI SS-OCTA that could image additional areas of non-perfusion. In particular, we obtained in the same order wider imaged with EFI SS-OCTA, then FA and lastly with SS-OCTA and consequently larger areas of non-perfusion with EFI SS-OCTA, then FA and lastly with SS-OCTA. At the same time, we have to consider that the use of EFI technique can potentially overestimate the non-perfusion areas due to an over-representation of the edges of the images where the curved surface is projected flat.

Another factor that should be taken into account is the different resolution of the images obtained. In fact, EFI SS-OCTA cover larger area then SS-OCTA without EFI with the same number of A-scan and resulting in a lower resolution of the image [14]. This different resolution could explain the findings of larger areas of non-perfusion and the lower valued of VD and FD.

**Fig 3:**
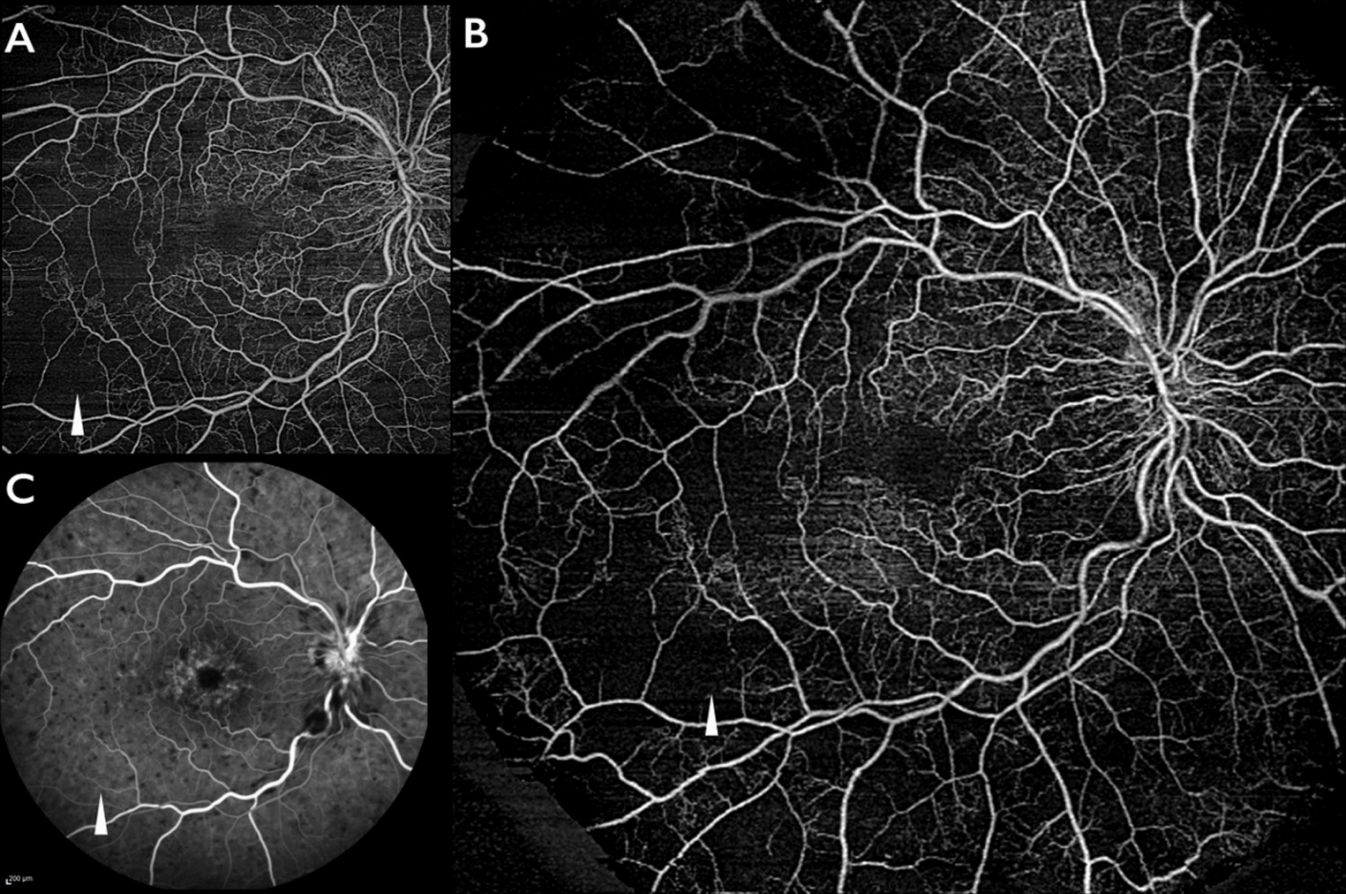
Swept-source optical coherence tomography angiography (SS-OCTA) and fluorescein angiography (FA) images of one representative eye with retinal vein occlusion. SS-OCTA without and with EFI (A and B) showing a similar distribution of non-perfused areas but not corresponding to FA (C). Interestingly, OCTA images display an interrupted vessel (white arrow) whereas FA apparently displays a complete filling of the same vascular trunk (white arrow). The analysis of FA early phases revealed slow but compete filling suggesting that the velocity of blood flow influences the OCTA signal thus possibly leading to an over estimation of non-perfusion.

Other factors that could influence the analysis are blood flow velocity and possible errors in the segmentation boundaries. Particularly slow flow may result in black signal misinterpreted as non-perfusion in OCTA and not corresponding to FA (Fig 3). However, in our study we used the preset “retina” segmentation to reduce artifacts secondary to segmentation failure.

Our results are in agreement with previous studies that investigated the EFI SS-OCTA with standard FA [9,11]. Hirano et al. [9] documented in patients with diabetic retinopathy that EFI SS-OCTA captured larger areas of the fundus compared to SS-OCTA without EFI and FA. However, they did not find significant differences between the two imaging modalities on the extension of non-perfusion areas and number of new blood vessels. In particular, the average extension of EFI SS-OCTA was inferior to our study since they presented cropped rectangular images while in our series we used a prototype lens designed specifically by Zeiss to perform EFI examination and to avoid the positional instability of the lens. Moreover, we also evaluated the branching complexity of the capillary network with the FD that showed significantly correlations between VD and areas of non-perfusion explaining the changes in the perfusion of the retina.

Differently, Kakihara et al. [11] evaluated the degree of ischemia in eyes affected by retinal vein occlusion concluding that EFI SS-OCTA provided larger images of retinal vasculature compared to FA and with an accurately resolution of retinal capillary non-perfusion. For this reason, the authors suggested that EFI SS-OCTA is very useful imaging modality for the evaluation of retinal ischemia in eyes with RVO. However, the comparison of retinal non-perfusion areas between SS-OCTA and FA needs more considerations. As presented in Fig 3, EFI SS-OCTA and SS-OCTA without EFI clearly indicate areas of capillary non-perfusion that are not appreciable in FA. This is related to the respective limitations of the two imaging techniques. OCTA is influenced by the velocity of flow in which a slow flow inside the vessels resulting in black signal misinterpreted as non-perfusion as shown in Fig 3. On the other side, in FA the leakage of the dye from the vessels may mask the capillary non-perfusion resulting in an underestimate evaluation.

The present study has several limitations as the small number of included eyes. Moreover, we have to consider that other factors may have influenced the results of our analysis as the poor pupil dilation or media opacities. At least, in our series we included a limited subset of pathologies and with only a retinal involvement.

In conclusion, we found that a single capture of EFI SS-OCTA covers a larger area of the retinal vasculature than conventional FA. Moreover, the use of EFI SS-OCTA in the clinical practice can save a lot of time, as several OCTA images have to be made for one montage and may burden the cooperation of the patient. Despite, the determination of retinal ischemia seems to be easier and more accurate using EFI SS-OCTA, FA offers more details of the perfusion status of the retina as it is not influenced by the velocity of the flow. At this moment, FA should be considered as the gold standard for the evaluation of retinal perfusion. Furthers studies are needed on the comparison between FA and OCTA to clearly assess the condition of diseases affecting the retinal vasculature in more peripheral regions.

## Supporting information

S1 File Data set. (XLSX)
